# To Work or Not to Work, That Is the Question: The Psychological Impact of the First COVID-19 Lockdown on the Elderly, Healthcare Workers, and Virtual Workers

**DOI:** 10.3390/healthcare9121754

**Published:** 2021-12-18

**Authors:** Silvia Andreassi, Silvia Monaco, Sergio Salvatore, Gaetano Maria Sciabica, Giulio De Felice, Elena Petrovska, Rachele Mariani

**Affiliations:** 1 Department of Dynamic and Clinical Psychology and Health Studies Sapienza, University of Rome, 00185 Rome, Italy; silvia.monaco@uniroma1.it (S.M.); sergio.salvatore@uniroma1.it (S.S.); gaetanomaria.sciabica@uniroma1.it (G.M.S.); rachele.mariani@uniroma1.it (R.M.); 2 Xenophon College London, University of Chichester, Chichester PO19 6PE, UK; giulio.defelice@uniroma1.it; 3 Derner School of Psychology, Adelphi University, Garden City, New York, NY 11530-0701, USA; petre813@newschool.edu

**Keywords:** coronavirus pandemic, emotional regulation, post-traumatic growth, narratives, referential activity

## Abstract

The spread of COVID-19 created a state of emergency all over the world and played a big role in the decline of the mental health of citizens. The context of the workplace became an important variable in the impact of the lockdown on individuals. In this study, we deepened the categories of healthcare workers (HWs), virtual workers (VWs), and the elderly, along with their emotional approach to this emergency. A sample of 257 participants (ElderlyN = 62; HWsN = 104; VWsN = 91) completed: a semi-structured interview on their experience during lockdown via telephone; an online survey with a sociodemographic questionnaire; the Difficulties in Emotional Regulation Scale (DERS); and the Post-Traumatic Growth Inventory (PTGI). Linguistic measures of the Referential Process were utilized to code the interviews. An independent ANOVA explored the variability among groups. The results show more affective language in the Elderly (M = 0.0310, SD = 0.0070) and a growth in spirituality (M = 4.16, SD = 3.17). HWs displayed a higher PTGI (M = 56.84, SD = 20.29), while VWs displayed a lower PTGI (M = 50.02, SD = 21.05). Moreover, VWs presented higher scores in Impulse on the DERS (M = 11.67, SD = 5.05) and a more cognitive/abstract narration (Reflection IREF M = 0.0260, SD = 0.0071; Reorganization IWRRL M = 0.5419, SD = 0.0032; Referential Activity IWRAD M = 0.4978, SD = 0.0029). This study aims to take the work context into consideration to create focused interventions.

## 1. Introduction

The COVID-19 pandemic, which has become the most important public health crisis of our time, has led to heavy and unique worldwide repercussions. The general population of each country has experienced the emotional, social, and economic impact of this emergency. In Italy, as for many other countries, the lockdown imposition changed the lifestyle of each citizen, regardless of their age or social condition. Research demonstrated the consequences this condition provoked in the mental health of the general population, which involved new psychological symptoms [1] or a worsening of pre-existing pathological conditions [2]. When the research became saturated with this data, scholars then focused on understanding the reason behind this collective mental health emergency and explored the differences and cognitive mechanisms that led (or could have led) to psychological concerns. 

One of the most interesting social contexts regarding this theme is the lockdown experience, imposed by 9 March 2020 until 4 May 2020. According to research, the lockdown led to an increase in mental illness, when compared to previous or subsequent data [3,4]. This context of restrictions led to changes in the daily routine and in the social context through different aspects, such as loneliness and financial concerns [5]. However, the impact of these aspects is dependent on the different categories of the population and could therefore produce altering psychological responses. According to research, the workplace context can be considered an important factor that modulated the impact of lockdown on the mental health of individuals [6].

Healthcare workers (HWSs) represent the people in front-line positions during the COVID-19 pandemic, especially during the lockdown phase [7]. They were reputed to be the group at the highest risk of contracting the virus [6]. Therefore, this led to a specific emotional response, such as the fear of bringing the contagious virus home and spreading it to their loved ones [8]. Research explored the emotional consequences of COVID-19 on HWSs, showing high levels of burnout among these workers [9] and, in particular, high levels of emotional exhaustion [10]. The amount of work and time pressure also led to serious difficulties in emotional regulation for these workers [11]. These data appear even more worrisome if we consider the importance of emotional labor required of HWSs and the necessity of the appropriate emotional regulation [12]. Moreover, recent research highlights how the use of appropriate emotional strategies seems to be a protective factor for the consequences of COVID-19 on the mental health of HWSs [13], therefore also aiding Post-Traumatic Growth [14]. 

The majority of those who did not work in a healthcare context and were spared from the numerous firings of this socio-economic crisis adapted their work to a digital modality, becoming “virtual workers” (VWs). In Italy, the term “smartworker” is colloquially utilized to define this category of workers. This remote modality produced an overload of work and an increased pressure to perform for VWs [15]. One of the most recurrent practices of this new way of working is holding meetings via videoconference. This working practice produced a particular condition, commonly referred to as “Zoom fatigue,” characterized by cognitive exhaustion [16]. One of the explanations for this phenomenon, which is different from the normal fatigue resulting from typical meetings, is the lack of non-verbal communication in this kind of meeting, which required more cognitive work, especially for the listener [17].

Finally, the elderly population faced many problems of their own. Italy features the largest elderly population in Europe [18] and over 107,935 of its citizens aged 70 and over lost their lives (https://www.epicentro.iss.it/coronavirus/sars-cov-2-decessi-italia. Accessed in data 1 November 2021). Other facets of the problems faced by the older population during lockdown included physical inactivity [19], loneliness, and isolation [20]. However, this category experienced positive effects of the lockdown imposition [21], such as a reduction in emotional distress [22] and a higher level of general well-being [23] when compared to younger citizens. 

Multiple Code Theory (MCT) could be utilized to better understand the various strategies developed to face a similar trauma [24]. The MCT focuses on the complexity of emotional functioning and includes three systems for processing and representing emotional information: the nonverbal sub-symbolic (visceral sensations related to an arousal contition); the nonverbal symbolic (symbols); and the verbal symbolic (words). In processing emotions, the most important step occurs between the sub-symbolic and the symbolic non-verbal systems [25], when symbols are created. According to this theory, the Referential Process is a particular process that links these three processing systems. Due to this system, information shifts from a nonverbal form to an image and then, finally, to language [26]. However, RP works in a bidirectional way: verbal material can also be translated into a non-verbal form. The RP can be defined by three principal phases: emotional arousal, narrating/symbolizing, and reflecting/reorganizing. According to MCT, traumatic conditions can disturb an appropriate work of RP, thereby creating a disconnection of the three systems [27,28], that lead to symptomatic manifestations. This theoretical frame led to the ability to trace the connection or integration between symbolic and subsymbolic systems, within narratives, therefore highlighting the levels of integration and elaboration of emotional responses. 

The MCT research is usually referred to clinical settings and applied to understand linguistic differences among different psychopathologies [29]. Recently, MCT was applied through linguistic measures to understand the impact of COVID-19 in the general population as well. RP linguistic measures highlighted that people used different functions in processing and integrating such a collective traumatic experience [30], where significant mechanisms of defense emerged [31], and were also connected to psychological symptoms [32], according to MCT’s disconnection suppositions.

The COVID-19 lockdown created new challenges for each social category, which activated new responses, such as the significant adaptation of older people; or symptoms, such as burnout for healthcare workers; and Zoom fatigue for virtual workers. In this sense, different psychological strategies of adaptation could be found in each social category. More specifically, we hypothesize that:

1. The three categories of people (healthcare workers, virtual workers, and the elderly) used different emotional regulation strategies, with different post-traumatic development skills and different narrative processes.
2. Emotional regulation is affected by the methods of coping with the traumatic experience of lockdown and with the symbolization processes.

## 2. Materials and Methods

### 2.1. Participants

The sample was recruited from the general Italian population through snowball sampling. A total of 257 individuals, all of whom resided in central Italy, participated in the study and all the data from the participants were included in the analysis according to adherence with the interview questions. The number of people included in the study was based on previous studies and an a priori power analysis performed using G_Power 3.1.9.2 (UCLA, Dusseldorf, Germany; available online: https://www.psychologie.hhu.de/en/arbeitsgruppen/allgemeine-psychologie-und-arbeitspsychologie/gpower. accessed on 10 December 2021). It was estimated that 252 people (total sample) would be enough to detect differences between the three groups with an effect size (f) of 0.25 and a power of 95%, assuming an α.05. A further power analysis was performed to retrospectively estimate whether the size of the sample enrolled possessed sufficient power to perform a linear regression model analysis with six predictors. It was estimated that 215 people (total sample n) would be enough to perform this analysis with an effect size (f) of 0.10 and a power of 95%, assuming an α.05.

The Elderly (N = 62) group was composed of 33 females and 29 males, with a mean age of 71.7 (SD = 9.2). The average amount of years of education in this category was 11.23 (SD = 5) and 25.3% of the participants identified as being in a stable romantic relationship during the time of the study.

The VWS (N = 91) group was composed of 41 females and 50 males, with a mean age of 29.86 (SD = 5.55). In this group, the average amount of years of education was 17.07 (SD = 5.25) and 34.1% identified as being in a stable romantic relationship.

The HWS (N = 104) group was composed of 68 females and 36 males, with a mean age of 35.26 (SD = 10.5). In this group, the average amount of years of education was 17.07 (SD = 5.25) and 34.1% identified as being in a stable romantic relationship.

Throughout the entire process of this research, no participants dropped out of the study.

### 2.2. Procedure

The research project was presented during lectures to university students of psychology (given the lockdown period, the courses were held remotely). The students were asked whether they knew healthcare workers, virtual workers, or elderly people who could participate in the research. An email and a telephone contact were provided for interested parties to contact within a deadline of 10 days.

During the first telephone call, the details of the research were discussed and if the individual agreed to participate in the study, an appointment was made. All the subjects joined during the last period of the hard lockdown and completed the interview in the first month of partial reopening. The data collection for the research was carried out between 15 May 2020 and 15 June 2020, which was close to the reopening following the total lockdown of the previous three months.

The interviews were carried out by five researchers. The interviewer was mandated to complete specific training on the conduct of interviews prior to interviewing the participants.

The participants were initially asked to provide informed consent through a link sent out by the researchers. Once individuals formally consented to participating in the study, they were asked to fill out a preliminary information survey online regarding personal and demographic data, along with two other questionnaires (see Section 2.3). This first part lasted about 15 min. After completing the questionnaires, the study required a fully recorded semi-structured interview, conducted via telephone, regarding the experience of confinement. The interview lasted about 30–40 min.

This study guaranteed the privacy and anonymity of each participant. The participants were warned that they could stop their participation at any time during the interview and the overall study. Moreover, the interviewer reserved a brief moment after the interview to thank and reassure the participant. 

After the data collection, three groups were created from the total sample according to their occupational category (virtual worker, health care worker, or elderly person). Each participant was then assigned to the relevant group. The assignments were performed through an observational naturalistic method.

### 2.3. Measures

The Italian Discourse Attributes Analysis Program (IDAAP) and the linguistic measures of the referential process, otherwise known as The IDAAP system [33], was designed to read texts and compare them word by word. The IDAAP refers to MCT and it applies different dictionaries [34] at the same time, thereby producing derived measures and covariations between the two dictionaries. In this study, we used the following dictionaries and derived measures for the Italian language:

- The Italian Weighted Referential Activity Dictionary (IWRAD) is a dictionary consisting of 9596 words and represents the computerized measure of RA. It features a scale ranging from 0 to 1. High scores represent a high level of referential activity. The most frequent words with low IWRAD weights scores are associated with a subjective focus rather than pointing to external objects and describing situations, in the present rather than the past tense. For a deeper discussion, see [33].
- The Italian Reflection Dictionary (IREF) is a dictionary composed of 908 abstract words concerning how people think and communicate their thoughts. It includes basic logic words and cognitive or logical words.
- The Italian Sensory Somatic Dictionary (ISensD) is a dictionary composed of 1926 words related to the body and bodily activities. The number of ISensD words in a speech sample is a measure of the sub-symbolic activation.
- The Italian Sum Affect Dictionary (ISAffD) is a dictionary consisting of 1786 words concerning how people feel and how they communicate their feelings. It indicates an emotional response, positive or negative. The ISAffD is composed of three sub-dictionaries: positive affect (IAffP); negative affect (IAffN); and neutral affect without a specific valence (IAffZ).
- The Italian Weighted Reflection and Reorganization List (IWRRL) refers to the reorganization and reflection function, where a speaker attempts to recognize and understand the emotional value of an event or set of events. It contains a list of 1633 Italian words and high scores on this measure represent high reflection/reorganization. It concerns a person’s reasoning related to an experience that has been vividly experienced.
- The Italian DisFluency Dictionary (IDFD) is a small set of words, as well as repeated words, incomplete words, and filled pauses, that people tend to use when struggling to communicate. High scores typically characterize the arousal phase in which emotional schemas are activated.
- Difficulties in Emotion Regulation Scale (DERS) [35]. The DERS is a 36 item self-report on a 5 point Likert scale (from 5, “Almost always”, to 1, “Almost never”) that measures emotional regulation. The self-report questionnaire provides a total score (DERS TOTAL), indicating the general level of difficulty in emotional regulation (total score range = 36–180) and six scores corresponding to several facets of emotion dysregulation capacities. The six subscales of emotional regulation are:
- The Non-Acceptance scale, which concerns the non-acceptance of the emotional response. It is made up of six items that reflect the tendency to experience negative secondary emotions in response to one’s own primary negative emotion, as well as the subject’s difficulties in accepting the negative emotion or discomfort that this causes.
- The Goals scale, which indicates the difficulty in engaging in a behavior directed towards a goal and is represented by five items that reflect the difficulty in completing one’s work or concentrating when experiencing negative emotions.
- The Strategies scale, which concerns limited access to one’s own regulation strategies. The eight items reflect the level of confidence the person has in his or her ability to manage and modulate negative emotions and the belief that little can be done to regulate emotions effectively once an individual is upset.
- The Impulse scale, which expresses the difficulty in controlling behaviors and includes six items that reflect the difficulty in maintaining control over one’s behavior when experiencing negative emotions.
- The Clarity scale, which concerns the difficulties in recognizing the emotion experienced and its five items reflect the degree to which a person recognizes the emotion they are experiencing.
- The Awareness scale, which expresses reduced emotional self-awareness and is expressed by six items that reflect emotional awareness, i.e. the degree of attention paid to one’s emotional state.

For the present study, the Italian adaptation of DERS was used [36,37].

The Posttraumatic Growth Inventory (PTGI) [38]. PTGI is a 21 item self-report on a 6point Likert scale ranging from 0 (no variation) to 5 (high variation) that measures the perception of positive change in response to a severe stressor. The self-report questionnaire provides a total score, indicating the general post-traumatic growth (PTGI TOTAL), with high scores indicating positive growth (total score range = 0–105) and five scores corresponding to five subscales. The subscales are:

- The Relationships With Others subscale, which concerns the development of a greater sense of compassion for or closeness with others, changes in behavior on an interpersonal level, the construction of new relationships, and the strengthening of previous relationships.
- The New Possibilities subscale, which evaluates the presence of changes in life goals, openness to new experiences, approaches to facing choices more consciously, and the new possibilities that are experienced when people develop a new life path that would not be possible if they had not experienced a crisis.
- The Personal Strength subscale, which evaluates a greater sense of self-confidence in facing the obstacles of life, a better acceptance of even unfavorable circumstances, and changes in the subject’s perception of their own identity.
- The Changes in Spirituality subscale, which measures personal growth associated with a better awareness of spiritual issues and an increase in sharing spiritual moments with others.
- The Appreciation of Life subscale, which concerns the individual’s re-evaluation of the value of daily life, changes in priorities, and the birth of new life values.
- The Italian version of the PTGI used for this study [39], shows excellent total internal reliability (Cronbach’s alpha = 0.93), and an acceptable-to-high internal reliability for the five factors (alpha interval Cronbach’s from 0.74 to 0.86).

#### Semi-Structured Interview

To conduct this survey, an ad hoc semi-structured interview was used, consisting of 19 stimulus questions aimed at collecting narratives on the experience of confinement. The following areas were investigated: personal and family situation; emotions and thoughts about confinement; flashbulb memories; relationship with the media; routines, space–time organization, and re-organization in the context of confinement; fears related to confinement; coping strategies; recurring thoughts; changes in self, sleep, and dream activity; relationship changes; and relationships. A second part of the interview consisted of close-ended questions about: hours spent on social media; the individual’s cohabitant during lockdown; months of pre-lockdown cohabitation; and a question to be answered using a Likert scale (1 to 7) regarding their fear of being infected.

The interview was entirely audio-recorded and subsequently transcribed in full.

### 2.4. Statistical Analysis

The statistical analyses were conducted using the Statistical Package for Social Science (SPSS), Version 26 for Windows (IBM, Armonk, NY, USA). The data were reported as frequencies and percentages for discrete variables and as means and standard deviations for continuous variables. An ANOVA for independent groups was performed among the three groups (HWSs, VWs, and the Elderly), investigating the possible variability of psychological variables (DERS, PTGI, linguistic measures and questions from the second part of the interviews). Finally, we performed a linear regression analysis using global Emotional Regulation (DERS Total scale) scores as dependent variables and PTGI total, Symbolizing (IWRAD), Negative Affect, Disfluency, time dedicated to social media, and fear of being infected as independent variables

## 3. Results

### 3.1. Descriptive Analysis

A total of 257 (female = 142) participants, composed of 104 Healthcare Workers (mean age = 35.26, SD: 10.52); 62 elderly people (M = 71.47, SD: 9.02); and 91 virtual workers (M = 29.86, SD 5.55). The total amount of years of education held by the HWs averaged 16.98 (SD = 2.7); for the elderly it was 11.23 (SD: 5.00); and for VWs, it was 17.07 (SD: 2.25). Each group was also asked to report their relationship status: 40.6% of HWs reported being in a romantic relationship, as did 25.3% of Elderly people and 34.1% of VWs. Age was determined to be significantly positively correlated to PTGI Change in Spirituality (r = 0.238, *p* < 0.000) and negatively correlated to PTGI New Possibility (r = −0.131, *p* < −0.05). No other significant relationships emerged between age and other variables. No significant differences emerged among gender, years of education, and relationship status. 

### 3.2. Comparison among Groups: HWs, Elderly, and VWs

We hypothesized that the three categories of people used different emotional regulation strategies, with different post-traumatic development skills and different narrative processes. In analyzing the different groups of variables, we found the results described below. The comparison between the three groups through an analysis of ANOVA for independent groups highlighted the following results, for the specific questions of this study. Elderly people spent more time on social media networks than HWs (Bonferroni *p* < 0.05) and were also the group that had lived the longest with a significant other before the lockdown, compared to both HWs and VWs. Further, VWs presented a significant difference in their perception of the risk of contagion consistent with the reality of the pandemic, with respect to the HWs and the elderly (see Table 1).

Regarding the emotional dysregulation DERS scale, three strategies that were significant according to the ANOVA comparison (see Table 1). Specifically, the elderly had a greater distance from negative emotions; they reflected the tendency to experience negative secondary emotions in response to their negative emotions, or to experience reactions of non-acceptance with respect to their discomfort, compared to VWs (post-hoc Bonferroni *p* < 0. 05). Meanwhile, VWs displayed higher values for adopting goal-oriented behaviors, such as difficulties in concentrating and performing a task when experiencing negative emotions, as well as in adopting impulsive behaviors, compared to HWs (post-hoc Bonferroni *p* < 0.05).

Compared to the Post-Traumatic Growth measured with the PTGI scale, we found that the three groups did not differ in the total PTGI scale, which is the sum of the different strategies explored. However, specific differences emerged between the subscales: we found that there were two significant sub-scales (see Table 1): Personal Strength and Spirituality. Specifically, the HWs felt they possessed greater personal strength compared to the VWs (post-hoc Bonferroni *p* < 0.05), while the elderly showed significant higher scoring in growth of Changes in Spirituality, compared to the VWs and HWs (post-hoc Bonferroni *p* < 0.05). The VWs did not present any higher scoring in any specific measure on the PTGI subscale.

As for the linguistic analysis, the comparison between groups, with respect to the referential process, demonstrated different symbolic processes and emotional processing in the three specific groups according to ANOVA (see Table 1). The first set of data was related to the number of words spoken during the interview and demonstrated that HWs talked more about their history in relation to the pandemic. They were also less influenced by their story than VWs.

Specific results emerged regarding the use of the words used to express negative affect, and they differentiated the three groups. Of the three groups, the elderly demonstrated greater use of negative-affect language, followed by the HWs, and finally, the VWs (post-hoc Bonferroni *p* < 0.05). The elderly also displayed a greater use of positive-affect words and, in general, used more affective language than the other two groups. Finally, the VW group demonstrated a significantly greater use of intellectualized (IREF) and reflexive (IWRRL) words while their lower symbolization index (IWRAD) displayed a more cognitive and abstract narrative than the other two groups (post- hoc Bonferroni *p* < 0.05)

### 3.3. Regression Analysis

We hypothesized that post-traumatic growth strategies, along with maladaptive behaviors and the ability to narrate the pandemic experience, preceded emotional dysregulation. 

To test this hypothesis, we conducted a linear regression analysis (Table 2) using global Emotional Regulation (DERS Total scale) scores as dependent variables and the PTGI total, Symbolizing (IWRAD), Negative Affect, Disfluency, Time Dedicated to Social Media, and Fear of Being Infected as independent variables. 

The model explains 48% of the DERS total scale (adjusted *R*^2^ = 0.38; *F* = 6.75; *p* = 0.000), thus indicating an adequate fit of the model tested. The independent variables that showed a significant effect were: Fear of Being Infected (β = 0.31; *t* = 3.55; *p* < 0.01), PTGI Total (β = 0.29; *t* = 3.15; *p* < 0.01), Time Dedicated to Social Media (β = 0.17; *t* = 2.62; *p* < 0.01), and Disfluency (β = 0.16; *t* = 2.47; *p* < 0.01). Symbolizing (IWRAD) and Negative Affect did not show any statistically significant results.

## 4. Discussion

### 4.1. Peculiarities among Healthcare Workers, Virtual Workers, and the Elderly

Compared to our first hypothesis, which investigated the differences between the three groups, we can say that the results were consistent with the first hypothesis. The three groups, in relation to the different life experience, and the work versus non-work context, expressed different characteristics in dealing with the pandemic reality. The three groups did not differ on the Global Dysregulation and Post-Traumatic Growth scales; however, HWs showed lower levels of emotional dysregulation on the Goals and Impulses subscales. This result is consistent with their focus on work and the great commitment sustained during the pandemic, compared to the other two groups. By contrast, the VWs perceived the least danger of contagion but used more impulsive strategies and demonstrated more confusion with respect to the objectives. The elderly seemed consistent with lived reality, being the largest population affected by the virus. From these first results, the VWs appeared more disoriented with respect to danger and the strategies needed to face it. 

### 4.2. Work Setting as a Protective Factor

This same aspect is confirmed by the ability to learn from the traumatic experience. In fact, the elderly emphasized the spiritual dimension while the VWs displayed more of a personal ability to cope with the trauma. Both aspects are understandable considering the different contexts; while the elderly were required to receive greater protection for the higher-risk situation they were in, maximum operational participation was required of the HWs. In other words, work for the HWs featured an ego-syntonic function, which strengthened their abilities, despite the greater exposure involved. The VWs kept their work functions more distant from real activities to protect themselves; they were also more alien and maladaptive. The differences in narratives mirror these differences between groups. In fact, from the linguistic perspective, the VWs were more emotionally activated than the other groups, therefore expressing a greater diffusion which, unlike the HWs, did not activate a symbolized story and an experience about the pandemic, but instead, a more abstract and intellectualized discourse. In this way, they were perhaps trying to explain the strong disorientation that the sudden job change required. 

### 4.3. A New Way to Explore Emotional Dysregulation

The second hypothesis explored how the relationship between post-traumatic growth, narrative variables most relevant to the study, and some maladaptive behaviors could predict greater emotional dysregulation. The data confirmed the already existing research, in which a greater perception of risk [40] and greater dependence on social networks [41] seemed to be the main variables connected to emotional dysregulation. In relation to the second hypothesis, one innovative discovery is that of the linguistic variable, Dysfluency (which expresses the difficulty in finding words), and its ability to manifest itself as a variable connected to emotional regulation, and one of the predictor variables of emotional dysregulation. This result is supported by research that highlights how the difficulty in finding words, or the use of filler words (such as mm, um, like, etc.) is closely linked to emotional activation. Higher scores therefore express the need to regulate arousal [42,43]. Instead, the relationship between post-traumatic growth and emotional dysregulation is counterintuitive. Total PTGI is a predictor of emotional dysregulation, but contrary to our assumptions, the relationship is direct. In other words, those who used greater post-traumatic strategies did so in relation to greater emotional dysregulation. This linear relationship between post-traumatic growth and emotional regulation highlights how the use of functional strategies to deal with a traumatic event still requires emotional activation. In other words, the subjective ability to use an experience, albeit a traumatic one, seems to be linked to the way in which a person uses his or her emotional sphere. Therefore, the ability to use specific coping strategies to cope with emotional dysregulation seems to be the real trauma resilience factor.

## 5. Conclusions

The goal of this research was to study the impact of total confinement in three different populations: VWs, HWs, and the elderly. Each group had to face a radical reorganization of their life. The elderly found themselves closed off at home and isolated from habitual and familial relationships; the HWs were required to work at an unprecedented level of urgency, in a health emergency; while the VWs found themselves having to remain confined to their homes and adapting to continuing work from home.

This study showed how the psychological adaptation strategies to stress implemented by the three groups were different from each other and how the three categories of people used different strategies for regulating emotions, different post-traumatic development skills, and different narrative processes.

It seems that the group struggling the most following the lockdown was that of the VWs. In fact, VW appears to have been the social category with the most difficulty in contacting their emotions, favoring narrative strategies centered on intellectualization and abstraction, displaying difficulty concentrating and greater impulsiveness.

We do not think that virtual work can be the sole cause of this difficulty, but that this study has identified an unexpected aspect that merits further research.

The difference between the three groups is essentially given by the internal–external dimension. HWs maintained a working exterior and a private life interior. Even the elderly maintained this difference between the inside and outside with the uniqueness of finding themselves only inside, with a foreclosed outside. In these two groups, the boundary between inside/outside was maintained: a private interior versus a public exterior.

The VWs found themselves in a completely different situation, by having a very short amount of time to experience an unchosen overlap and a collapse of the boundary between internal and external. We believe that the collapse of the inside/outside boundary may be the variable that differentiated the adaptive strategies and difficulties presented by the three groups. Although these differences were probably more evident in the first total lockdown, they are not necessarily stable. When virtual work becomes a stable way of working over time, we assume that the people involved automatically restore emotional boundaries between inside/outside. We aim to deepen these observations with further research.

## 6. Limitations

The results of this study need further confirmation with larger cohorts for each group (VWs, HWs, and the elderly). It would be also interesting to complete a follow-up study with the present research in order to observe possible differences in emotional response, two years after the lockdown imposition. As mentioned above, it is possible that VWs became more confident in remote work conditions and would therefore present with different data. The final important limitation is the lack of information regarding possible different settings in the VWs group, such as the number of hours spent remotely working and the tools used.

## Figures and Tables

**Table 1:** ANOVA independent group comparison of HWs, elderly, and VWs.

	HWSs		Elderly		VWSs			
	Group 1		Group 2		Group 3			
	N = 104		N = 62		N = 91			
Questions	Mean	SD	Mean	SD	Mean	SD	F	*p*
Time Dedicated to Social Media	1.95	0.81	2.40	1.65	2.15	1.65	3.24	0.041 *
Cohabitant During Lockdown	1.62	1.32	1.48	1.30	1.66	1.09	0.39	0.680
Fear of Being Infected	4.54	1.60	4.48	5.24	3.05	1.11	7.60	0.001 **
Months of Pre-Lockdown Cohabitation	10.75	9.41	41.05	10.80	9.07	9.35	163.20	0.000 **
DERS	Mean	SD	Mean	SD	Mean	SD	F	*p*
Nonacceptance	11.89	5.06	13.89	6.56	11.60	5.27	3.536	0.031 *
Goals	10.63	3.56	11.90	4.59	12.01	4.10	3.335	0.037 *
Impulse	10.01	3.63	10.68	4.69	11.67	5.05	3.292	0.039 *
Awareness	14.47	4.36	15.69	4.32	15.11	4.55	1.493	0.227
Strategies	14.20	5.48	16.11	6.20	14.90	5.84	2.075	0.128
Clarity	10.52	3.96	10.98	4.53	11.32	4.49	0.840	0.433
DERSTOTAL	71.72	18.02	79.34	22.36	76.24	20.03	2.986	0.052
PTGI	Mean	SD	Mean	SD	Mean	SD	F	*p*
Relationship With Others	18.06	8.05	17.34	8.49	15.70	7.89	2.065	0.129
New Possibilities	13.25	5.36	11.42	5.61	13.16	5.75	2.426	0.091
Personal Strength	12.22	5.24	10.32	5.51	9.90	4.95	5.245	0.006 **
Changes In Spirituality	3.12	1.97	4.16	3.17	2.92	1.91	5.849	0.003 **
Appreciation For Life	10.18	3.67	9.85	3.82	8.90	3.88	2.854	0.060
PTGITOTAL	56.84	20.29	53.10	23.07	50.02	21.05	2.445	0.089
RP Linguistic Measures ^1^	Mean	SD	Mean	SD	Mean	SD	F	*p*
Words	2716.66	1476.58	2173.03	1494.19	2027.18	973.49	7.229	0.001 **
MDF	0.094	0.042	0.096	0.046	0.109	0.040	3.616	0.028 *
IAffN	0.0131	0.0037	0.0150	0.0051	0.0110	0.0035	18.422	0.000 **
IAffP	0.0108	0.0032	0.0126	0.0044	0.0108	0.0033	5.512	0.005 **
IAffS	0.0277	0.0061	0.0310	0.0070	0.0251	0.0055	17.315	0.000 **
IAffZ	0.0038	0.0022	0.0035	0.0021	0.0033	0.0017	1.496	0.226
IRef	0.0246	0.0061	0.0228	0.0071	0.0260	0.0071	4.131	0.017 *
ISenS	0.0425	0.0072	0.0398	0.0099	0.0353	0.0070	20.592	0.000 **
IWRAD	0.5007	0.0030	0.5009	0.0039	0.4978	0.0029	25.626	0.000 **
IWRRL	0.5431	0.0027	0.5431	0.0029	0.5419	0.0032	4.699	0.010 *

* *p* < 0.05; ** *p* < 0.01; ^1^ IWRAD = Italian Weighted Referential Activity Dictionary; IWRRL = Italian Weighted Reflection and Reorganization List; IRef = Italian Reflection Dictionary; IDFD = Italian Disfluency Dictionary; ISenS = Italian Sensory-Somatic Dictionary; MDF = Italian Disfluency Dictionary; ISAffN = Italian Dictionary of Negative Affects; ISAffP = Italian Dictionary of Positive Affects; ISAffS = Italian Dictionary of Sum of Positive, Negative, Neutral Affects; ISAffZ = Italian Dictionary of Neutral Affects.

**Table 2:** Model derived from the linear regression analysis.

	Beta	t	Sign.
PTGI TOTAL	0.29	3.15	0.002
Time Dedicated to Social Media	0.17	2.62	0.009
Fear of Being Infected	0.31	3.55	0.001
MDF	0.16	2.47	0.009
IAffN	0.057	0.92	0.357
IWRAD	0.043	0.68	0.499

## Data Availability

Data available on request due to privacy restrictions.
